# Development of a blueprint for plant-derived hospital meals: a multidisciplinary co-creative study

**DOI:** 10.1007/s00394-026-04040-5

**Published:** 2026-06-30

**Authors:** M. A. van Bree, H. M. Kruizenga, B. C. Schouten, M. R. Soeters

**Affiliations:** 1 Nutrition and Dietetics, Amsterdam Gastroenterology Endocrinology Metabolism (AGEM), Amsterdam UMC, University of Amsterdam, Meibergdreef 9, Amsterdam, Netherlands; 2 Endocrinology & Metabolism, Internal Medicine, Amsterdam Gastroenterology Endocrinology Metabolism (AGEM), Amsterdam UMC, University of Amsterdam, Meibergdreef 9, 1105 AZ Amsterdam, Netherlands; 3 Nutrition and Dietetics, Amsterdam Movement Sciences (AMS), Amsterdam UMC, University of Amsterdam, De Boelelaan 1117, Amsterdam, Netherlands; 4 Communication Science, Amsterdam School of Communication Research (ASCoR), University of Amsterdam, Nieuwe Achtergracht 166, Amsterdam, Netherlands

**Keywords:** Protein transition, Hospital meals, Plant-derived protein, Hospitalized patients

## Abstract

**Purpose:**

Hospitals increasingly seek to align patient meals with sustainable food policies while ensuring nutritional adequacy and patient acceptance. However, the limited availability of palatable high-protein plant-derived meals with complete amino acid profiles hampers implementation. This study aimed to develop and evaluate fully plant-derived hospital meals for nutritional adequacy, palatability, cost-effectiveness, and feasibility within sustainable food policy frameworks.

**Methods:**

A multidisciplinary, co-creative study was conducted between April and December 2025 with hospital staff and industry partners. Meal development criteria were derived from clinical guidelines and hospital policies and prioritized by a multidisciplinary team. Recipes were iteratively developed and evaluated through cooking and tasting sessions, then optimized for nutritional composition, amino acid profile, and palatability. Nutritional values were calculated using the Dutch Food Composition Database, and protein quality estimated through weighted mean digestibility and aggregated amino acid scores across meal protein sources.

**Results:**

Thirty meal recipes were developed and combined into seven daily menus providing 90–97 g protein per day, primarily from soy, lentils, grains, and nuts. To increase protein in breakfast and lunch without enlarging portions, pea protein isolate and soy-based drinks were incorporated. Total daily menu costs ranged from €10.50 to €12.30.

**Conclusion:**

Fully plant-derived meals can provide adequate, palatable, and cost-effective protein, suitable for clinical use, although trade-offs between protein quality, taste, cost, and portion size remain. These meals offer a practical blueprint for hospitals seeking to adjust animal-to-plant protein ratios and support the transition toward sustainable nutrition.

**Supplementary Information:**

The online version contains supplementary material available at 10.1007/s00394-026-04040-5.

## Introduction

Global sustainability goals such as the Planetary Health Diet and European Green Deal [1–3] along with new governmental policies [4, 5], require a shift towards healthier, more plant-derived nutrition. More specific, under the Dutch government’s ‘Green Deal Sustainable Healthcare’, hospitals are expected to achieve a 40/60 animal-to-plant protein ratio by 2030, while some healthcare institutions have set even more ambitious targets, aiming for predominantly or fully plant-derived food offerings [6]. Implementation of this transition in clinical practice remains challenging, particularly for patient nutrition [7, 8]. One major barrier is the nutritional adequacy of plant-derived meals, especially for protein quantity and quality [9]. According to the European Society for Clinical Nutrition and Metabolism (ESPEN) guidelines, hospital meals must be energy-dense and rich in high-quality protein to support recovery and prevent protein-energy malnutrition [10]. Yet, current plant-derived meal offerings in hospitals rarely meet these nutritional standards [11, 12].

Compared to animal protein, individual plant-derived protein sources generally provide less protein, have a lower digestibility, and contain a less favourable amino acid profile [13, 14]. The latter refers to the composition and amount of essential amino acids in a protein relative to human requirements [15]. This influences its capacity to support protein synthesis. A lower digestibility and suboptimal amino acid profile may reduce anabolic potential, which is concerning in clinical settings, where older or acutely ill patients often suffer from increased catabolism and anabolic resistance [16]. Consequently, there is a need for meals that can meet both sustainable food policy and the nutritional requirements during acute illness.

Development of suitable protein rich plant-derived meals with a complete amino acid profile, necessitates combining complementary protein sources such as grains and legumes. The essential amino acid lysine, important for tissue repair and immune function, is often the limiting amino acid in plant-derived meals [8, 17, 18]. Incorporating plant-derived ingredients naturally rich in lysine, such as lentils, chickpeas, soy, and almonds, can improve the amino acid profile. However, achieving a sufficient amount of lysine remains difficult especially for breakfast and lunch meals, as these ingredients are less commonly used during these meal times in Western diets.

Besides amino acid adequacy, required portion size may further limit practical implementation. For example, to reach 20 g of protein with a complete amino acid profile using whole food protein sources, portion sizes may need to increase substantially, sometimes exceeding meal volumes by 25% [19]. These larger portion sizes can be problematic for hospitalized patients, who often experience reduced appetite due to illness or treatment-related symptoms such as nausea, vomiting, altered gastrointestinal motility, fatigue, or pain. Fortification with high-quality plant-derived protein powders such as soy, pea or potato protein may offer a potential solution to increase protein density without expanding portion volume [14].

Alongside protein quantity and quality, palatability is also an important factor for patient acceptance and implementation. Our previous work has shown that taste strongly influences patients’ intention to consume more plant-derived nutrition [20]. Plant-derived meals are often perceived as less tasty or satisfying [21–23]. Factors such as flavour, texture, aroma and presentation, which can collectively be referred to as culinary success factors (CSFs), may support the development of more appealing meals and improve patient acceptance [24]. In addition to taste, economic considerations are important, as the transition to healthier and more sustainable nutrition can have varying financial impacts. For example, plant-based meat and dairy alternatives may increase costs, whereas meals based on whole foods can potentially reduce them [25, 26].

Although previous studies have explored plant-derived meals in healthcare settings, these have generally focused on isolated aspects such as protein quality [17, 27], nutritional composition [28–31], food waste [32, 33], or patient acceptance [20, 34, 35]. To our knowledge, no studies have simultaneously addressed protein quality, portion size, palatability, cost, and operational feasibility within a multidisciplinary co-creative meal development process. Therefore, practical guidance for implementing nutritionally adequate plant-derived hospital meals in clinical practice remains limited.

To address these practical challenges in the implementation of more plant-derived nutrition for patients this study used a multidisciplinary, co-creative approach to develop fully plant-derived meals suitable for clinical use. We integrated insights from current literature, company based culinary expertise and food service practice, with a specific focus on protein quality and quantity, portion size and taste. The findings of this study provide ready-to-use menus and practical insights for future meal development and implementation in hospital food programs, thereby supporting the protein transition in the healthcare sector. Moreover, these menus may serve as a blueprint and can be adapted by hospitals to achieve their desired animal-to-plant protein ratios, facilitating the broader shift toward plant-derived nutrition in clinical practice.

## Methods

### Study design and stakeholder collaboration

This co-creative study was conducted between April and December 2025. A co-creative approach was chosen to actively involve stakeholders, create a sense of ownership, and integrate diverse expertise, to enhance the practical applicability and likelihood of successful implementation of the meals [36]. As part of this process, a multidisciplinary expert group on plant-derived nutrition was established to design fully plant-derived meals that would meet clinical nutritional standards, align with patients’ taste preferences, and are practically feasible. The expert group consisted of chefs from both Amsterdam UMC (*n* = 2) and collaborating companies (*n* = 4), dietitians (*n* = 3), a medical doctor (*n* = 1), hospital facility service staff (*n* = 2), and company product- and R&D specialists (*n* = 4). Together, they selected the most important pre-defined meal criteria, developed and tested recipes and analysed the taste and nutritional value of the meals. Based on this, ready to use plant-derived meals were established for the general adult hospitalized population receiving standard hospital care. The meals were not designed for patients requiring enteral or parenteral nutrition, intensive care treatment, or texture-modified diets.

### Meal development criteria

For the development of the meals, the following pre-defined criteria were selected by the expert group that were based on literature [10, 15, 37–41] and current hospital policy of a large academic hospital in the Netherlands: a high protein content with a complete amino acid profile, good palatability, appropriate portion sizes, realistic costs, and practical feasibility. The amount of protein per menu was based on ESPEN guidelines for hospital nutrition (1.2 g/kg body weight and ~ 20 EN% protein) and set at a minimum of 85 g per day to address the protein requirements of the average patient [10]. To meet these requirements, nutrient targets were also defined per meal. Main meals were designed to aim for ≥ 20 g of protein to support muscle protein synthesis, while in-between meals were targeted to provide ≥ 5 g of protein per serving [37–39]. A complete amino acid profile was defined as having all essential amino acids scores (AAS) ≥ 1 according to the Food and Agriculture Organization (FAO) amino acid scoring pattern for adults (mg/g protein) [15]. In line with FAO/WHO recommendations, protein quality scores ≥ 0.75 were used as a priori design criterion and considered indicative of ‘good’ protein quality, generally meeting human requirements for essential amino acids [42]. For other macro- and micronutrients, the desired minimum amounts were defined based on the Dutch dietary guidelines and the ESPEN micronutrient guideline [40, 41].

**Fig. 1:**
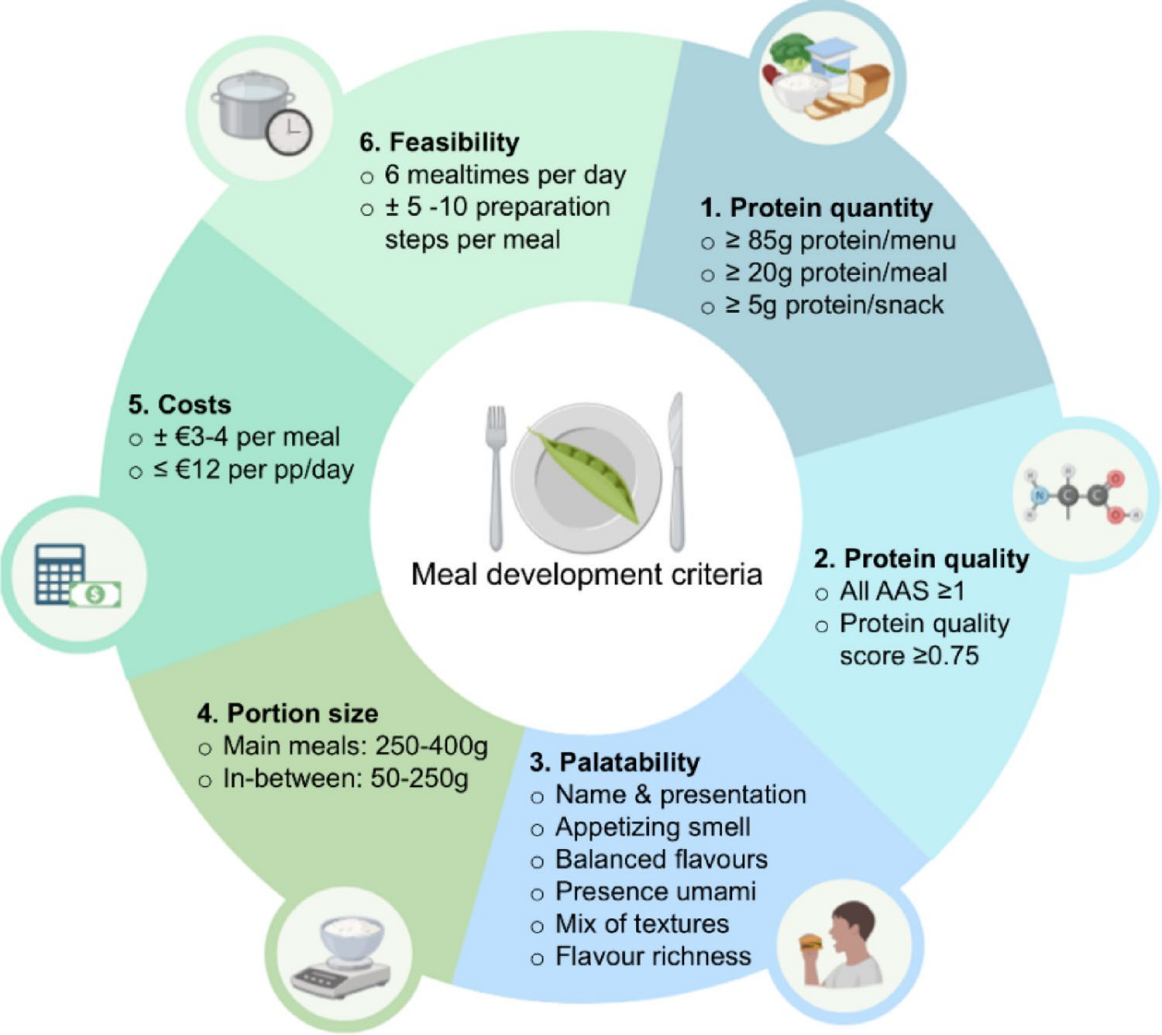
Plant-derived meal development criteria. This figure illustrates the six predefined criteria that guided the development of the meals: protein quantity, protein quality, palatability, portion size, cost, and feasibility. Protein quality was assessed based on amino acid composition and digestibility, with all essential amino acid scores required to be ≥ 1.0. In addition, an overall protein quality score (reflecting amino acid adequacy combined with digestibility correction factors) was required to be ≥ 0.75 to ensure sufficient protein quality for clinical use. AAS, amino acid scores

Palatability was defined using the Culinary Success Factors (CSF) [24], a framework which consists of six criteria to evaluate meals: (1) alignment of name and presentation with expectations, (2) an appetizing and appropriate aroma, (3) a balanced flavour profile, (4) the presence of a rich savoury flavour (umami), (5) a mix of textures in mouthfeel, and (6) overall flavour richness. Each dish was intended to meet at least five of the six CSF criteria. Portion size criteria were based on standard hospital serving practices, with target ranges of 250–400 g for breakfast, lunch, and dinner meals, and 50–250 g for in-between meals. If the amount of protein could not be reached within these portion sizes, protein fortification was used to meet both the criteria for protein and portion size. Costs were aligned with the hospital’s average daily food procurement budget of approximately €12 per patient per day. Finally, practical feasibility was defined in terms of minimizing preparation steps, with the intention of keeping meal preparation as straightforward as possible. As a general guideline, this corresponded roughly to no more than five steps for breakfast or lunch and no more than ten steps for dinner meals. An overview of all meal development criteria is presented in Fig. 1.

### Meal development and evaluation

Following the pre-defined meal criteria, 40 plant-derived recipes were created. Whole grains, minimally processed foods, and dishes inspired by a variety of global cuisines were incorporated wherever possible. Because of the importance of taste in meal acceptance, hedonic tasting sessions were conducted to assess the sensory qualities of each dish. Two three-hour sessions were required to prepare and evaluate all dishes. The recipes were prepared by chefs, after which all expert group members (*n* = 16) rated each dish separately for taste (flavour balance and overall richness) and texture (mouthfeel variety) on a 1–9 scale (1 = dislike extremely, 5 = neither like nor dislike, 9 = like extremely). Based on these evaluations, the 30 most promising dishes (score ≥ 7) were selected for further refinement. In a third cooking session, these recipes were re-prepared and modified to meet nutritional targets, essential amino acid profiles, appropriate portion sizes, and the remaining CSF criteria (alignment of name and presentation with expectations, aroma, and umami). Excluded meals, with reasons for exclusion are presented in Supplementary Table 3.

#### Calculation of nutritional value and estimated protein quality scores

Nutritional composition was calculated using the Dutch Food Composition Database (NEVO table, 2023) [43]. Amino Acid Scores (AAS) were calculated with the AminoFit-tool [19], based on amino acid composition data obtained from publicly available food composition databases (Danish Frida Food Database [44], FoodData Central [45], and MEXT [46]), and supplemented with data from literature [47].

Protein quality at meal level was estimated by integrating amino acid composition and requirements per gram of protein (AAS) derived from AminoFit with the weighted protein digestibility (wPD) of the protein sources in the meal (see formulas below). Because true ileal (gut) digestibility (TID) values for individual amino acids were not available for all protein sources, a general protein digestibility factor, based on previously published estimates, was assigned per ingredient at the product-group level [27]. Digestibility of processed plant-derived products (e.g. meat alternatives and dairy alternatives) were classified according to their primary protein source and processing category. Weighted protein digestibility (wPD) per meal was calculated as the sum of the digestibility of each protein source weighted by its proportional contribution to total meal protein. An overview of the protein digestibility factors per ingredient and wPD values are presented in Supplementary Table 2.

To calculate an overall protein quality score for each meal, the AAS and wPD were incorporated into a formula conceptually similar to the Protein Digestibility-Corrected Amino Acid Score (PDCAAS), as a composite proxy combining amino acid composition-based adequacy and protein digestibility. Specifically, the meal’s estimated protein quality was determined by multiplying the wPD by the aggregated lowest Amino Acid Score across all protein sources (AAS_meal_):

1 $$ \begin{aligned} {\mathrm{Estimated}}\;{\mathrm{Quality}}_{{{\mathrm{meal}}}} & = {\mathrm{Digestibility}}\left( {{\mathrm{wPD}}} \right) \\ & \quad \times {\mathrm{Lowest}}\;{\mathrm{Amino}}\;{\mathrm{Acid}}\;{\mathrm{Score}}\;({\mathrm{AAS}}_{{{\mathrm{meal}}}} ) \\ \end{aligned} $$

where the weighted mean digestibility (wPD) was calculated as the sum of the digestibility of each protein source (PD_i_), weighted by its proportional contribution (R_i_) to total meal protein:

2 $$ {\mathrm{wPD}} = \sum\limits_{{\mathrm{i}}} {{\mathrm{(R}}_{{\mathrm{i}}} \times {\mathrm{PD}}_{{\mathrm{i}}} )} $$

For each essential amino acid (y), the weighted essential amino acid content of the meal (EAA_y, meal_) was calculated as the sum of the proportional contribution (R_i_) of each protein source multiplied by its essential amino acid content per gram of protein (EAA_y, i_):

3 $$ {\mathrm{EAA}}_{{{\mathrm{y}},\;{\mathrm{meal}}}} = ~\sum {\mathrm{i}} \;\left( {{\mathrm{R}}_{{\mathrm{i}}} ~ \times ~{\mathrm{EAA}}_{{{\mathrm{y}},{\mathrm{i}}}} } \right) $$

The amino acid score of the meal (AAS_meal_) was calculated for each essential amino acid (*y*) as the ratio of its weighted content in the meal (EAA_y, meal_) to the corresponding adult reference requirement (EAA_y, ref_), with the lowest ratio across all amino acids (MIN_y_) defining the limiting amino acid:

4 $$ {\mathrm{AAS}}_{{{\mathrm{meal}}}} = {\mathrm{MIN}}_{{\mathrm{y}}} \left( {\frac{{{\mathrm{EAA}}_{{{\mathrm{y}},{\mathrm{meal}}}} }}{{{\mathrm{EAA}}_{{{\mathrm{y}},{\mathrm{ref}}}} }}} \right)~ $$

Finally, each recipe was analysed for the number of preparation steps and associated costs to evaluate practical feasibility. Costs were estimated based on ingredient prices obtained from the hospitals food ordering system (period: September - December 2025) [48].

#### Interpretation of the meal protein quality score

The estimated protein quality score is a study-specific proxy metric combining amino acid adequacy and protein digestibility at meal level. It should not be interpreted as equivalent to PDCAAS or DIAAS. The score is expressed on a ratio scale, where higher values indicate a more favourable combination of essential amino acid adequacy and protein digestibility. A value of 1.0 indicates that the meal meets the reference amino acid pattern after correction for digestibility. Values below 1.0 indicate lower estimated protein quality relative to the reference pattern, whereas values above 1.0 indicate that the combination of amino acid adequacy and digestibility exceeds the reference level. Unlike PDCAAS, the score is not truncated and may therefore exceed 1.0.

## Results

### Insights meal development process

A total of 30 plant-derived recipes were developed, comprising recipes for seven breakfasts, seven lunches, seven dinners and nine in-between meals. These were combined to create seven daily menus. Table 1 shows the recipes along with the applied criteria. As it proved nearly impossible to meet all pre-defined criteria for each meal without compromising protein content, taste, preparation steps, or portion size, meals that did not meet all criteria were nevertheless retained within the overall daily menus. In particular, breakfast and lunch meals did not reach the minimum of 20 g of protein per serving. For several lunch dishes, the protein content was increased by supplementation with a plant-derived dairy alternative, such as a soy dessert or a glass of soy drink. Because portion sizes sometimes made the latter impossible, certain breakfast and in-between meal items were further fortified with a pea protein isolate. This applied, for example, to smoothies, overnight oats, and the date and almond protein bar. The choice of type and amount of protein isolate was primarily guided by taste. Because soy was already widely used as a protein source in most of the dishes, alternative commercially available protein isolates were tested, including potato (Sosa, Catalonia, Spain), fermented pea protein (Mattisson, Växjö, Sweden) and vanilla flavoured pea protein (XXL Nutrition, Deurne, Netherlands). Potato and fermented pea protein isolates were considered too strong and less palatable. Therefore, the flavoured pea protein isolate was selected as the best alternative to use in the dishes.

#### Evaluation culinary success factors

Plant-derived ingredients were generally associated with less aroma by the expert group, particularly in breakfast recipes such as quark bowls and overnight oats. In contrast, for savoury dishes, vegan cheeses could function as strong flavour contributors. Vegan parmesan exhibited a more intense aroma than young-style vegan cheese. While their pronounced aroma could enhance flavour perception in lunch and dinner dishes, excessive use occasionally resulted in an overpowering taste. Overall strategies to enhance aroma in the sweeter dishes included roasting fruits (e.g., apples) and nuts (e.g., walnuts and hazelnuts) or adding spices such as cinnamon, as applied in the breakfast recipes. Texture and mouthfeel were mainly influenced by ingredient selection: oats provided structure, and oat flour was preferred over wheat flour (e.g. in breakfast pancakes) due to its higher protein content and whole-food profile. Nutritional yeast enhanced flavour and contributed modestly to protein content, although it also affected dish colour, as observed in the chicory casserole. Moreover, fruits, seeds, and nuts added crispness or crunch, further improving mouthfeel. Vegetables and fruits contributed to visual appeal and meal presentation, for example in sandwiches, vegetable omelettes, and smoothies. Finally, meal names were adapted to clearly reflect the main ingredient, aligning consumer expectations with presentation.

**Table 1:** Meal assessment

	Meals	Energy (kcal)	Protein (g)	Digestible protein (g)	Weight (g)	Costs (€)	Preparation steps
	*Breakfast*						
1.	Soy based quark bowl	351	18	16	270	2,71	2
2.	Banana peanut butter sandwich + soy drink	649	27	23	420	1,15	2
3.	Overnight oats	365	18	15	250	1,47	3
4.	Blueberry smoothie	237	20	19	255	2,23	1
5.	Hazelnut pancakes + mango smoothie	529	16	15	350	1,98	8
6.	Almond and apple sandwich + soy drink	585	25	21	380	1,47	2
7.	Soy quark with fruits and muesli	358	15	13	250	1,65	2
	*Lunch*						
8.	Sandwich with chickpea and paprika spread + apple cinnamon dessert	572	20	17	304	2,33	3
9.	Spinach pie with soy mince	343	17	14	250	2,25	5
10.	Sweet potato and bean salad + soy drink	370	29	23	295	1,62	5
11.	Sandwich with pea spread + apple cinnamon dessert	546	23	19	340	2,21	2
12.	Mediterranean tomato soup + soy drink	328	23	20	280	3,16	5
13.	Poke bowl	556	19	16	320	4,31	5
14.	Veggie omelette with roasted pepper spread + soy drink	402	23	20	220	2,70	5
	*Dinner*						
15.	Eggplant pilaf with bean cassoulet + seasonal fruits	513	29	24	585	3,77	5
16.	Chicory casserole with Dutch-style soy beef stew	539	25	21	410	3,80	13
17.	Vegetable quiche + seasonal fruits	558	23	19	530	4,18	5
18.	Green lentil risotto	474	34	29	400	4,01	5
19.	Lentil pasta pesto with soy mince	643	33	26	350	3,47	7
20.	Chickpea curry + fruit salad with soy yoghurt	615	24	19	602	2,90	10
21.	Vegetable Besengek (Indonesian stew)with rice + seasonal fruits	490	27	24	515	4,78	9
	*In-between meals*						
22.	Carrot banana cake	271	6	4	70	2,71	5
23.	Waffle with lentil spread	135	6	4	55	0,56	5
24.	Date and almond protein bar	142	8	8	50	0,63	6
25.	Fruit salad with soy yoghurt dip	131	6	6	135	1,49	2
26.	Mango smoothie	122	10	10	160	1,25	1
27.	Strawberry smoothie	107	10	10	160	1,10	1
28.	Lupin-based egg muffin	266	10	7	95	1,03	7
29.	Apple cinnamon dessert	152	7	7	130	1,04	7
30.	Strawberry vanilla dessert	94	5	5	130	1,17	5

### Nutritional value weekly menu

**Fig. 2:**
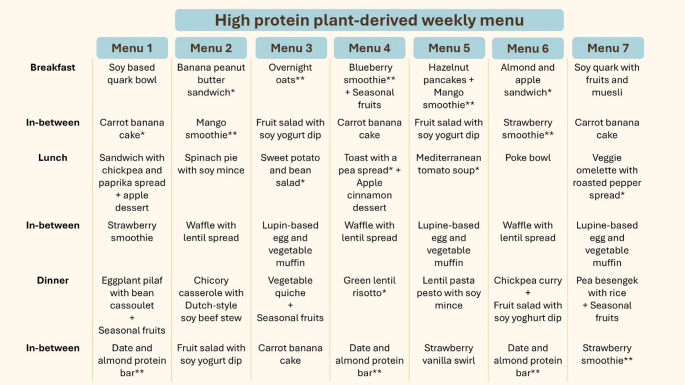
Plant-derived menu. *Indicates meals supplemented with a glass of soy drink. **Indicates meals fortified with pea protein isolate

Figure 2 shows the seven daily menus. According to the hospital’s food program, the created menus were structured around six daily moments: breakfast, lunch, dinner and three in-between meals per day. The total protein content of the menus ranged from 90 to 97 gram per day, of which 77 to 84 gram was estimated to be digestible protein. Fats contributed between 37% and 42% of total energy intake (EN%), largely due to the inclusion of nuts, seeds, and flavourings such as soy cooking cream and vegetable oils. The contribution of saturated fats did not exceed the recommended upper limit of 10% of total energy in any of the menus (Table 2).

Dietary fibre content varied from 39 to 54 gram per menu. None of the menus achieved the recommended intake for calcium, whereas only one menu met the recommended intake for vitamin B12. Sodium content remained below the recommended maximum of 2400 mg per day in all menus. Detailed information on individual products and recipes, including their nutritional values, are available in Supplementary Table 1.

**Table 2:** Nutritional value daily menus

	Menu 1	Menu 2	Menu 3	Menu 4	Menu 5	Menu 6	Menu 7	Menu criteria
Energy kcal	2025	1920	1960	1900	1990	2140	1900	
Protein g (EN%)	97 (19)	91 (19)	90 (18)	97 (20)	93 (19)	91 (17)	90 (19)	≥ 85 g/d
Digestible protein g	84	77	74	83	79	77	77	
Fat g (EN%)	88 (39)	88 (41)	82 (37)	82 (39)	93 (42)	96 (40)	90 (42)	20–40 EN%
Saturated fat g (EN%)	16 (7)	22 (10)	23 (10)	21 (10)	20 (9)	21 (9)	21 (10)	≤ 10 EN%
Carbohydrates g (EN%)	188 (37)	170 (35)	189 (30)	168 (36)	177 (36)	205 (38)	150 (34)	> 40 EN%
Fibre g	47	40	54	48	39	46	45	> 30 g/d
Sodium mg	1922	1989	1743	1840	2274	1990	1615	< 2400 mg/d
Potassium mg	3870	4610	5600	4200	4242	3232	4636	≥ 3500 mg/d
Calcium mg	874	632	823	690	727	693	749	> 950 mg/d
Iron mg	18	18	24	19	20	17	21	11 mg/d
Vitamin C mg	150	64	67	88	90	75	70	≥ 75 mg/d
Zinc mg	10	8	13	11	9	11	14	> 7 mg/d
Vitamin B12 µg	2	1.8	1.6	1.9	2.4	1.5	1.6	> 2.4 µg/d
Total weight g	1640	1500	1640	1505	1610	1570	1500	
Price €	11,65	10,50	10,60	11,50	12,30	10,97	12,05	≤ €12

EN%, energy percentage

### Evaluation of estimated meal protein quality

Table 3 presents the distribution of protein sources, the lowest Amino Acid Score, the weighted protein digestibility, and the resulting protein quality score per meal. Each meal combined multiple protein sources to optimize the essential amino acid profile. Wheat, peanuts, oats, lentils, pea protein isolate, and soy protein contributed most to the total protein content per meal. All meals included some soy protein to improve both protein quantity and quality. The individual AAS values of all meals were above 1, indicating that the amount of each essential amino acid met the FAO adult reference pattern prior to digestibility correction. Overall meal protein quality scores were above 0.75.

**Table 3:** Meal protein combinations

	Meals	Protein sources and contribution to total protein content (%)	Lowest AAS (EAA)	wPD	Protein quality score
	*Breakfast*				
1.	Soy based quark bowl	Soy (57%), oats (15%) almond (25%)	1.2 (met + cys)	0.85	1.02
2.	Banana peanut butter sandwich + soy drink	Wheat (31%), peanut (35%) cashew (8%), soy (26%)	1.0 (lys)	0.85	0.85
3.	Overnight oats	Oats (31%), soy (42%), pea isolate (27%)	1.5 (leu)	0.88	1.32
4.	Blueberry smoothie	Soy (31%), pea isolate (60%), chia seed (9%)	1.6 (met + cys)	0.96	1.53
5.	Hazelnut pancakes + mango smoothie	Oats (26%), soy (37%), pea isolate (37%)	1.5 (met + cys)	0.90	1.35
6.	Almond and apple sandwich + soy drink	Wheat (32%), almond (41%), soy (27%)	1.0 (lys)	0.85	0.85
7.	Soy quark with fruits and muesli	Soy (63%), walnut (16%), oats (11%), sunflower seeds(10%)	1.3 (lys)	0.86	1.12
	*Lunch*				
8.	Sandwich with chickpea- and paprika spread + apple cinnamon dessert	Wheat (43%), chickpea (25%), soy (32%)	1.1 (lys)	0.88	0.96
9.	Spinach pie with soy mince	Soy (58%), lentil (24%), wheat (18%)	1.3 (met + cys)	0.89	1.15
10.	Sweet potato and bean salad + soy drink	Lentil (55%), white beans (18%), soy (27%)	1.1 (met + cys)	0.80	0.88
11.	Sandwich with pea spread + apple cinnamon dessert	Wheat (38%), pea (29%), soy (33%)	1.1 (met + cys)	0.87	0.96
12.	Mediterranean tomato soup + soy drink	Kidney bean (12%), soy (69%), wheat (19%)	1.3 (met + cys)	0.91	1.18
13.	Poke bowl	Rice (16%), soy (39%), soy-bean (26%), lentil (19%)	1.2 (met + cys)	0.82	0.98
14.	Veggie omelette with roasted pepper spread + soy drink	Wheat (36%), lupin (32%), soy (32%)	1.1 (lys)	0.86	0.95
	*Dinner*				
15.	Eggplant pilaf with bean cassoulet	Rice (10%), soy (48%) legumes (43%)	1.3 (met + cys)	0.84	1.10
16.	Chicory casserole with dutch-style soy beef stew	Soy (80%), potato (8%), walnut (12%)	1.2 (met + cys)	0.89	1.06
17.	Vegetable quiche	Soy (53%), lentil (24%), wheat (23%)	1.3 (met + cys)	0.89	1.15
18.	Green lentil risotto	Lentil (48%), soy (45%), mushroom (7%)	1.1 (met + cys)	0.83	0.91
19.	Lentil pasta pesto with soy mince	Lentil (53%), soy (34%), cashew (13%)	1.1 (met + cys)	0.81	0.90
20.	Chickpea curry + fruit salad with soy yoghurt	Chickpea (38%), lentil (21%), rice (14%), soy(27%)	1.2 (met + cys)	0.79	0.95
21.	Vegetable besengek with rice	Green bean (7%), rice (13%), soy (80%)	1.2 (met + cys)	0.89	1.07

AAS, amino acid score; EAA, essential amino acid; wPD, weighted mean digestibility

### Costs

The costs of individual ingredients ranged from €0.10 for items such as vegetables, beans, and legumes to €2.44 for soy-based meat alternatives. Meal costs varied by type: €0.83–€2.71 for breakfast, €1.62–€4.32 for lunch, and €2.90–€4.78 for dinner meals. Daily menu costs ranged from €10.50 to €12.30. Within these ranges, higher meal costs were mainly associated with the use of protein-rich ingredients such as soy-based meat and dairy alternatives, protein isolates and nuts, which were necessary to meet the minimum protein requirements per meal.

## Discussion

### Discussion of main results

This study aimed to develop protein-rich, plant-derived meals suitable for clinical use through a pragmatic, multidisciplinary co-creative approach. Collaboration among chefs, dietitians, physicians, industry partners, and hospital facility services resulted in seven complete plant-derived menus that meet clinical standards, including high protein content and an adequate amino-acid profile. Achieving these targets proved particularly challenging due to the relatively low protein density of plant-derived ingredients, especially in breakfast and lunch meals, whereas dinner meals more consistently met these requirements. As a result, developing 100% plant-derived meals that are both nutritionally adequate and palatable required navigating unavoidable trade-offs, such as increased portion sizes or the use of protein isolates. The overall process showed the complexity of balancing protein density, flavour, portion size, and budget constraints, and underscored the value of a multidisciplinary co-creative approach to achieve feasible solutions.

Meal development was guided by the principle of using healthy, minimally processed plant foods, such as legumes and whole grains, which support long-term health and sustainability goals, including reduced environmental impact and a lower risk of chronic diseases [3, 49–51]. Their use in clinical settings, however, is more challenging, as nutritional requirements during acute illness differ substantially from those in healthy populations [10, 41, 52, 53]. Inadequate protein intake is associated with muscle loss and adverse clinical outcomes, including prolonged hospital stay, impaired wound healing, increased postoperative complications, and reduced quality of life [54, 55]. Although minimally processed plant-derived foods are recommended in plant-centred diets, reliance on these foods alone showed a compromised protein adequacy due to the lower protein density. In practice, we found that processed plant-derived products, such as soy-based meat and dairy alternatives, were essential to increase total protein content and also to provide micronutrients such as calcium and vitamin B12. As such, these products allowed elevated protein requirements to be met without increasing portion sizes. At the same time, because they generally contain more sodium and deviate from whole-food principles, their contribution may be greater to environmental sustainability than to overall health benefits.

Soy-based products were frequently used to improve both protein quantity and quality of the meals. Soy has been extensively studied, with occasionally conflicting findings regarding its health effects. On the one hand, soy provides isoflavones with antioxidant properties that have been associated with reduced cancer risk and alleviation of menopausal symptoms [56–59]. On the other hand, isoflavones can bind to oestrogen receptors (ERα and ERβ) and act as tissue-specific oestrogen modulators, which has led to caution in patients with hormone-sensitive cancers and individuals with thyroid disorders [60–62]. Current clinical evidence indicates that even relatively high isoflavone intakes (40–100 mg/day) do not adversely affect hormone levels, thyroid function, or clinical outcomes in healthy individuals or in patients with breast or prostate cancer [58, 63–66]. In the present menus, the average soy protein content was 33 g/day, contributing approximately 20–40% of total protein intake. As no international upper intake level has been established and Dutch dietary guidelines do not specify maximum soy intake [67], adverse effects at this level are unlikely. Nevertheless, a more balanced distribution of protein sources may be desirable. Increasing legumes or whole grains would increase meal volume, while adding nuts and seeds would substantially raise fat intake. Therefore, incorporating limited amounts of dairy could help reduce reliance on soy while maintaining protein quality and remaining consistent with sustainable dietary frameworks such as the EAT-Lancet Planetary Health Diet.

Soy, nuts, and seeds were often used in the meals and represent relevant food allergens. Nevertheless, allergies to these foods are relatively uncommon in the general population (< 1%) [68, 69]. For patients with nut or seed allergies, alternative plant protein sources such as legumes and soy can be used. For patients with soy allergy, this is more challenging in the context of achieving adequate protein intake, and alternative protein sources such as pea protein isolate or potato protein isolate may be considered. However, their use may be limited by palatability and therefore requires further evaluation in clinical practice. More broadly, the present meals were designed to reflect a broad hospital population; however, substantial heterogeneity exists in hospital patients, particularly regarding appetite levels and dietary restrictions. While the meals provide a structured framework for meeting nutritional requirements, individual preferences and appetite should guide intake in patients with reduced appetite, even when this includes animal-derived options. Medical and dietary restrictions (e.g. post-gastrointestinal surgery diets, phosphate restriction, or iron deficiency management) should always take precedence over adherence to plant-derived nutrition, even when these meals are carefully designed.

Combining multiple plant protein sources improved amino acid profiles. While lysine, methionine, and cysteine were the lowest-scoring amino acids, all essential AAS exceeded 1. The relatively low lysine and methionine and cysteine content observed is consistent with previous studies [47, 70, 71]. These findings indicate that, even with good combinations, achieving optimal protein quality in fully plant-derived meals remains challenging, especially when elevated protein needs and limited meal volumes must be considered. In addition to protein, vitamin B12 and calcium sufficiency was a challenge in our developed menus. Prolonged inadequacy of vitamin B12 may lead to megaloblastic anaemia and neurological complaints [72], whereas insufficient calcium intake may compromise bone health and increase fracture risk which is particularly relevant in older and immobilized patients [73, 74]. Therefore, implementation of fully plant-derived menus in clinical practice requires calculating meal composition, on both protein and micronutrients and, if necessary, the use of fortified products or supplementation.

Fibre content of the developed menus was above the recommended daily intake of 30–40 gram as advised by the Dutch Health Council [75]. A higher fibre intake is associated with a reduced risk of cardiovascular disease, overall mortality, pancreatic cancer, and diverticular disease [76]. These beneficial effects are largely explained by the ability of dietary fibre to slow glucose absorption by increasing intestinal viscosity and delaying gastric emptying, thereby improving postprandial glycaemic control, insulin sensitivity, and lipid metabolism [77]. In addition, dietary fibre lowers serum cholesterol and LDL concentrations by binding bile acids, stimulating their excretion, and increasing the conversion of cholesterol into new bile acids [78]. Moreover, fibre plays an important role in modulating the gut microbiota, leading to the production of short-chain fatty acids (SCFAs), which exert anti-inflammatory effects and contribute to a lower risk of chronic inflammation and cardiovascular mortality [79].

From a cost perspective, plant-derived protein sources were generally less expensive than animal-based alternatives. In line with previous studies [25, 26], higher costs were mainly driven by the use of meat alternatives. Variations in menu costs reflected differences in ingredient type, portion size, and the use of fortified products. Overall, total weekly menu costs remained within hospital budget limits, indicating that nutritionally adequate plant-derived menus can be implemented without increasing costs.

### Limitations and suggestions for further research

A strength of this study is its novelty in developing fully plant-derived meals for clinical use through a multidisciplinary co-creative approach, enhancing both nutritional adequacy and practical feasibility. Within this collaboration, each stakeholder contributed distinct expertise: chefs provided knowledge on flavour combinations and recipe development; dietitians were responsible for calculating recipes and assessing nutritional adequacy, including protein and amino acid content; facility services monitored cost control and evaluated feasibility regarding procurement and implementation within the healthcare setting; and industry partners contributed expertise in recipe and meal development as well as knowledge on innovative plant-derived products. However, several limitations should be noted. First, although the co-creative study design allowed to integrate diverse expertise and to enhance the practical applicability, a limitation of this method is the robustness of the data. In addition, protein quality estimates relied on generalized protein digestibility factors, as standardized amino acid-specific ileal digestibility data were not available for many plant-derived protein sources. Consequently, the reported protein quality scores should be interpreted as proxy estimates combining amino acid adequacy and protein digestibility rather than as DIAAS-equivalent protein quality scores. To assess the robustness of these estimates, a sensitivity analysis was performed using a 10% reduction in digestibility factors for all protein ingredients. This resulted in only minor changes in protein quality scores, with all meals remaining above the predefined threshold for sufficient protein quality (≥ 0.75), supporting the robustness of the study conclusions. More sophisticated methods, like tracer studies, could provide more precise insights into bioavailability and metabolic use of these plant-derived meals, including effects of the food matrix, and ‘true’ digestion rates. Second, we only focused on meal development; patient sensory evaluation and actual intake were not assessed. Therefore, acceptance and adequacy in real-world clinical settings remain uncertain. Intervention studies should evaluate patient acceptance and determine whether patients can actually meet their individual nutritional requirements with these menus. Finally, cost estimates were based solely on ingredient prices. Therefore, these prices reflect institutional purchasing rates and do not include additional costs such as preparation time, labour, or waste. As procurement conditions and contract pricing vary across institutions, these estimates should be interpreted as context-specific rather than universally generalisable.

## Conclusions

This study demonstrates that fully plant-derived meals can provide adequate protein, be palatable, cost-effective, and feasible for clinical implementation. Nonetheless, balancing protein quantity and quality, taste, cost, and sustainability involves unavoidable trade-offs, particularly within fully plant-derived meals. The meals developed in this study enable hospitals to tailor the ratio of plant and animal proteins to their clinical and sustainability priorities, while keeping patient needs central. Future research should focus on real-world implementation, including patient acceptance, and actual intake in relation to personalized requirements in daily clinical practice.

## Supplementary Information

Below is the link to the electronic supplementary material.

Supplementary Material 1

Supplementary Material 2

Supplementary Material 3

## Data Availability

No datasets were generated or analysed during the current study The generated data used in this study can be found in the Supplementary Materials.
